# Book Review

**Published:** 1899-09

**Authors:** 


**Diet and Food, Considered in Relation to Strength and Power of Endurance, Training and Athletics.** By Alexander Haig, M.D. Pp. vi., 86. London: J. & A. Churchill. 1898.—Dr. Haig's views on uric acid are now well-known, but the world has not yet sufficiently realised that "in diet lies the key to nine-tenths of the social and political problems that vex our nation and time." The universal experience of mankind for many centuries counts for nothing: "Diet, as at present used, is often the product of a vast amount of ignorance; it is the cause of a hideous waste of time and money; it produces mental and moral obliquities, destroys health and shortens life, and generally quite fails to fulfil its proper purpose." How to remedy all this, to show how diet may be easily made to fulfil its proper purpose, is the author's object in this little book, the work of an enthusiast in the subject and a radical reformer of diet both in health and disease.

**Wie ist die Fürsorge für Gemüthskranke von Aerzten und Laien zu fördern?** Von Prof. Dr. C. Fürstner. Pp. 64. Berlin: S. Karger. 1899.—This is a pamphlet dealing with some aspects of the care of the insane in Germany. The author advocates the formation of special departments in general hospitals for treatment of mental diseases (such as, for instance, exists at St. Thomas's Hospital), and then points out some legal difficulties which have arisen, in Germany, in the transference of patients from such clinics to asylums. He also discusses from what class nurses should be drawn, and recommends, as far as possible, the substitution of female for male nurses.

**Minutes of the General Medical Council for the Year 1898.** Vol. XXXV. London: Spottiswoode & Co. 1899.—No one, until he has looked through the various reports and recommendations of the numerous committees and branch councils, has any idea of the vast amount of work done by the General Medical Council. The public generally, and the medical profession in particular, ought to be most thankful that they are represented by such an able body of men, who at times may be a trifle too unprogressive and make some blunders, but who are altogether doing a very useful work.

**Aids to Examinations.** Part II. By T. Reuell Atkinson, M.D. Pp. 170. London: Baillière, Tindall & Cox. [n.d.].—If this book is really an "aid to examinations," so much the worse for the examinations. It is a curious medley of medicine, surgery, midwifery, pathology, anatomy, and physiology. No attempt has been made to arrange it in any order, so that the reader jumps from a question in medicine to another in anatomy. We recommend students to acquire their knowledge in a manner different from this, and not trust to such miscalled "aids."

**Epidemic Cerebro-Spinal Meningitis and its Relation to Other Forms of Meningitis: A Report of the State Board of Health of Massachusetts.** By Dr. W. T. Councilman, Dr. F. B. Mallory, and Dr. J. H. Wright. Pp. x., 5—178. Boston: Wright & Potter Printing Co. 1898.—This is a valuable contribution to the study of this disease, and gives the most complete account of the pathology. It deals with 111 cases of the recent epidemic in the New England States, and these were fully investigated. It is the more interesting as the true nature of the recurring epidemics of cerebro-spinal meningitis appears to have been first recognised in Massachusetts. Short accounts of the clinical history of each case are given, with, in many instances, charts of the pulse and temperature. The general result of this enquiry is to confirm the diplococcus of Weichselbaum as the cause of the disease; it was found in 31 out of 35 fatal cases in cultures taken from the exudations after death. Stress is laid on the importance of spinal puncture in diagnosis. This procedure gave a positive result in 38 out of 55 cases, and it is pointed out that from this source fresh light may be anticipated on the sporadic cases of the disease. The organism is not very viable outside the body; inoculation into animals was only once successful in producing meningitis, in the case of a goat. In the meningeal exudation large cells were found, often containing many leucocytes, and were derived from the cells of the connective tissue, and from those lining the lymph spaces. Amongst other changes in the brain, chiefly secondary to the meningeal inflammation, we notice the pronounced changes in the neuroglia, consisting of swelling and proliferation in the glia cells. All the changes in the brain-substance were very marked around the ventricles. The authors found that lesions of nerves were very common—of the cranial nerves, especially the second, fifth, and eighth;—and lesions of the spinal nerve-roots, varying in intensity, were found in every case in which they were examined. With regard to the important question of the distinction from pneumococcus meningitis, the authors say that without being warranted in making extensive generalisations, the absence or slight development of symptoms pointing to extensive infection of the meninges, of the cord and spinal roots, and extension of infection along the cranial nerves point to the pneumococcus infection. A series of good coloured plates of the most important bacteriological and pathological appearances is given, and the work affords the best account of the disease, derived from original observations, known to us, and has been admirably done in every respect.

**The Schott Treatment for Chronic Heart Diseases.** By Richard Greene. Second Edition. Pp. 16. London: The Scientific Press, Limited. 1898.—This brief account of the treatment of heart disease at Nauheim is of value to those who do not wish to buy a more expensive book. The number of patients visiting Nauheim continues to increase, so apparently the interest in the treatment is not materially abating.

**How to Avoid Tubercle.** By A. T. Tucker Wise, M.D. Pp. 15. London: Baillière, Tindall and Cox. 1898.—The size of this booklet shows what the power of condensation is able to effect. Dr. Wise emphasises the fact that many household pets, such as canaries and parrots, may be a source of tubercle dissemination, and considers that in regard to health a household is safer without any pets—except, perhaps, the cat and dog.

**A Synopsis of Surgery.** By R. F. Tobin. Pp. xx., 3—277. London: J. & A. Churchill. 1898.—This book, the author states, is a collection of leaflets of synopses of lectures delivered by him at St. Vincent's Hospital, Dublin. It is not illustrated, save for a few temperature charts; and the subject matter, which is interleaved with blank paper, is little more than a tabulation of facts "to lessen for the students the trouble of taking notes." For this purpose it will, no doubt, be useful, as the facts may be generally accepted and they are concisely stated. Externally the book resembles an *édition de luxe* of a devotional work.

**An Investigation on the Influence upon the Vital Resistance of Animals to the Micro-organisms of Disease brought about by Prolonged Sojourn in an Impure Atmosphere.** By D. H. Bergey, M.D. Pp. 10. Washington: The Smithsonian Institution. 1898. —The experiments here recorded were undertaken with a view to determining whether impure atmosphere produces detrimental effects upon the animal organism as shown in greater susceptibility to certain diseases. Owing to technical difficulties, results were obtained in only one series of experiments. In these it was shown that guinea-pigs living in an atmosphere containing a larger amount of carbonic acid than normal succumbed more quickly to tuberculosis when inoculated than did control animals.

**A Text-Book upon the Pathogenic Bacteria.** By Joseph McFarland, M.D. Second Edition. Pp. 497. London: Henry Kimpton. 1898. – The second edition of this work does not present many new features, the most important being additional chapters on whooping cough, mumps, yellow fever, hog-cholera, and swine-plague. The general scope of the volume remains very much as in the first edition, a favourable notice of which has already appeared in this *Journal.*

**A Synopsis of the British Pharmacopœia, 1898.** Compiled by H. Wippell Gadd. [Third Edition.] Pp. 183. London: Baillière, Tindall & Cox. 1898.—In this edition some fresh information is added, and the doses as expressed in metric equivalents are now given only approximately and not precisely exact; this arrangement, however, is much more practically useful. We can now repeat even more warmly the praise which we bestowed upon the first edition.

**Chloroform: Its Absolutely Safe Administration.** By Robert Bell, M.D. Pp. 40. Glasgow: Robert Love Holmes. 1898. —Whilst we entirely agree with Dr. Bell as to the desirability of using some form of graduated inhaler for the administration of chloroform, we doubt very much whether his pamphlet will in any way conduce to a general acceptance of his views. In a work avowedly written for and in the interests of the public, it is to be regretted that there should occur such a statement as the following: "Each death which occurs [in the administration of chloroform], to put it mildly, should in justice be described as one of culpable homicide." The statement, too, that in medical journalism there is an organised opposition to the facts of the chloroform controversy being known is without foundation. From a scientific standpoint this monograph is worthless, and it is calculated to do much harm by alarming and misleading the public.

**Diseases of the Skin.** By MALCOLM MORRIS. New [Second] Edition. Pp. xv., 589. London: Cassell and Company, Limited. 1898.—That there is a demand for this useful book is shown by this considerably enlarged edition. It seems almost superfluous to say that, upon the whole, the work is an excellent one for the busy practitioner as well as for the student. It is concise and clearly written. To some minor points only should we take exception. We have seen cases of "verruca necrogenica" amongst men who have had nothing to do "with handling dead tissue," and we are of opinion that one attack of erythema nodosum is, comparatively, rarely followed by a second one. Herpes zoster, in our opinion, is not such a right-sided affair as the author states ("the right side being far more often affected than the left," p. 158). We have investigated returns of our cases extending over nearly ten years, and out of 206 cases of zona the right side was affected in 100, the left in 93, and in 13 cases the incidence was unrecorded. We have seen a statement that zona occurs nineteen times on the right side to one on the left side of the body. On the other hand, amongst the older writers, Reil observes that he has always found it on the left half of the body. In looking over our statistics we do find sometimes a remarkable run, first upon one side and then upon the other; and we can well conceive that one observer, in the chapter of coincidences, might meet with a long one-sided succession. We have gone into this question before, and we believe the incidence would be, in a large number of cases, as much in favour of one side as the other. The index, printed in unnecessarily small type, is good, but has faults. "Rodent ulcer" should be p. 558, not 556; and on p. 138 we do not find a word about lichen planus. We congratulate the publishers on the improved external appearance of this edition.

**Essentials of the Diseases of the Ear.** By E. B. GLEASON, M.D. Second Edition. Pp. 206. London: Henry Kimpton. 1898.—We are not surprised that this little book has so rapidly reached a second edition. It contains in a small compass a large amount of useful information; and it is written in such a bright and easy manner that it forms a most readable work, notwithstanding the fact that it is arranged in the usually objectionable form of question and answer. The present edition contains a good account of the principal modern surgical procedures in intra-cranial complications of ear diseases; these are well illustrated and are plainly described. We know of no other small work on ear diseases to compare with this either in freshness of style or completeness of information.

**Transactions of the Clinical Society of London.** Vol. XXXI. London: Longmans, Green, and Co. 1898.—This volume contains a most interesting and varied collection of clinical reports on medical and surgical cases. Dr. Calot himself contributes a paper on his treatment of Pott's disease, and there are others—all more or less favourable to Calot's method—on the same subject. The report of the Committee on the antitoxin of diphtheria is also added, and the results of their investigation tend to show that by the use of antitoxin the general mortality is reduced by one-third.

**Transactions of the Royal Academy of Medicine in Ireland.** Vol. XVI. Dublin: Fannin and Co., Ltd. 1898.—A noteworthy paper in this volume is that on "Pneumonia: a Multiple Infection," by Dr. John W. Moore, who submits "that there is clinical evidence to show that a true pneumonitis may occur in . . . erysipelas, influenza, tuberculosis and enteric fever. Further, it is reasonable to suppose that in each case the pneumonitis is directly due to a localisation of the specific poison of the disease in the lung, whether that poison be a micro-organism itself or a toxin derived therefrom." Dr. W. R. Dawson, writing on membranous colitis, remarks: "The lesion is of the nature rather of a slight annoyance rather than a serious malady, and hence does not appear to demand any more energetic interference" than enemata, especially glycerine injections of about a couple of drachms, which appear to have been found most satisfactory. The "Clinical Report of the Rotunda Hospital" is, as usual, full of interest.

**Transactions of the Grant College Medical Society.** Bombay: The "Tattva-Vivechaka" Press. 1898.—These *Transactions* are for the most part a summary of a series of papers on the therapeutics of Indian drugs, of many of which we have never heard. The various meetings at which these papers were read must have been somewhat tame, but Dr. Dhargalker appears to be a host in himself, and scarcely any other topics were needed. Twenty or so new drugs at a meeting must give our Indian *confrères* abundant food for therapeutic investigation. The booklet is very unattractive in appearance.

**Transactions of the Iowa State Medical Society.** Vol. XVI. Burlington: The Keehn-Hafner Mfg. Co. 1898.—The Society whose transactions are here recorded has reached the 47th year of its existence. Many of the papers are on topics of general interest, and are worthy of a very wide circulation. The book would be all the better if more attention were given to the proofreading. We noticed "Kraska," "chololithiasis," "serum-theraphy."

**Transactions of the Medical Society of the State of New York.** Published by the Society. 1898.—The present issue contains the usual amount of valuable material which is to be found in these volumes, and is in every way equal to its predecessors. We would notice particularly the excellence of the articles on the use of the X-rays in medicine and surgery.

**Transactions of the Ohio State Medical Society.** Cleveland: J. B. Savage Press. 1898.—The Society contains 903 members and is evidently doing good work. The discussions on the various papers read at its fifty-third annual meeting are fully reported and are full of interest.

**Transactions of the South Carolina Medical Association.** Charleston: Walker, Evans & Cogswell Co. 1897.—This is the report of this Association's forty-seventh annual session. It shows that the South Carolinians are aspiring to be at all times up-to-date, and are working with the motto which their president wished them to keep continually before their eyes, "High endeavour and concerted action."

**Transactions of the American Surgical Association.** Vol. XVI. Philadelphia: William J. Dornan. 1898.—The most notable articles in this volume are on "The Etiology and Classification of Cystitis," by N. Senn; "The Question of Operative Interference in recent Simple Fractures of the Patella," by C. A. Powers; "The Use of Animal Toxins in the Treatment of Inoperable Malignant Tumors," by G. R. Fowler; and "A Clinical and Histological Study of Certain Adenocarcinomata of the Breast," by W. S. Halsted. Dr. Senn gives a most exhaustive account of his investigations into the subject of the various forms of cystitis, chiefly bacteriological, but partly also clinical, by which he proves that the essential or exciting cause of cystitis is invariably the presence of microbes in the tissues of the bladder. He describes the various forms of microbes met with, and discusses their mode of entrance into the bladder. The article well deserves a careful study. Dr. Powers has made a collective investigation among the Fellows of the American Surgical Association, the Members of the New York Surgical Society, and of the Philadelphia Academy of Surgery as to the advisability of operating on recent simple fractures of the patella, the form of operation preferred, and the number of cases operated on, with results. He gives a summary of the replies, and we are astonished at the conservative feeling manifested by the majority of these surgeons on the subject. Four would actually use Malgaigne's hooks in suitable cases where the fragments cannot easily be approximated. Seventeen are opposed to operation in any case. Nine would operate in all cases in which no distinct contraindication exists and in which the surroundings are satisfactory. Forty-one would operate on selected cases. Very high praise is given to the so-called "Dutch" method of treatment by massage. This is not half so well known in this country as it should be, and we refer our readers to this article for an admirable description of it. The article by Dr. Halsted contains his latest views and statistics on the methods and results of the supraclavicular operation and other operations for the removal of cancer of the breast, and we are appalled at the radical nature of the operation now advised by Dr. Halsted. The operation as now performed by him occupies from two to four hours and requires highly trained and skilful assistants. So much skin is removed that grafts have to be cut from the patient's thigh as large as or larger than one's hand to cover the raw surface. The neck is operated on in every case. Both pectoral muscles are removed. The axillary, subclavian and internal jugular veins are stripped clean. The supra-clavicular fossa is cleaned out. All fat is removed from the subscapular region and also from the supra- and infra-clavicular regions, while the nerves and vessels of the axillary cavity are stripped as clean as possible. After this extensive operation Dr. Halsted has met with an astonishing degree of success. One hundred and thirty-three cases have been operated upon; there have been only thirteen local and twenty-two regionary recurrences, while of seventy-six cases operated on more than three years ago, thirty-one are living without recurrence. This is far better than any results that have hitherto been shown, and it looks as if the day of the old operation had gone for ever.

**The Transactions of the Edinburgh Obstetrical Society.** Vol. XXII. Edinburgh: Oliver and Boyd. 1897.—Apart from the usual subjects of clinical interest which the *Transactions* always contain, there are in the present volume many papers on the speculative and scientific side of the subject, such as the question of the existence of a positive pressure in the growing pregnant uterus and the causation of twins. These are well worth reading, and will be found to contain many valuable suggestions.

**Transactions of the Ophthalmological Society of the United Kingdom.** Vol. XVIII. London: J. & A. Churchill. 1898.—In his Bowman Lecture, Mr. Priestley Smith, amongst many original observations, suggests that ordinary convergent strabismus, bearing as it does but little relation to the degree of the hypermetropia, is due to the "overaction of a motor centre which has escaped from conscious control." He lays stress on the necessity for "educative" treatment in addition to spectacles and operative measures. The elaborate report of the sub-committee on excision of the eyeball and the various operations which have been substituted for it occupies more than seventy pages. On the whole, the verdict is in favour of simple excision, with the addition in suitable cases of the insertion of a glass globe into Tenon's capsule. Mr. Gunn's very able paper on the ophthalmoscopic evidence of arterial disease is the outcome of an immense amount of careful observation. The diagnostic value of the conditions he depicts and describes, especially perhaps in cases where there is *no* albuminuria, is only just beginning to be recognised. In his paper on epithelial xerosis of the conjunctiva, Mr. Sydney Stephenson concludes that the disease is not usually due to the so-called xerosis bacilli, which are invariably present and are microscopically indistinguishable from diphtheria bacilli, but is caused by the irritation of spring and summer sunlight acting on the conjunctiva of anæmic and ill-nourished children. Upwards of a dozen cases are related of the successful detection of foreign bodies in the globe or orbit by the aid of the X-rays, and some good reproductions are given of Mr. Mackenzie Davidson's skiagrams.

**The Transactions of the Society of Anæsthetists.** Vol. I. London: The Medical Publishing Company, Limited. 1898.—The transactions of this infant Society deal chiefly with debatable points. Attention may be directed to Mr. Walter Tyrrell's suggestion to add ether vapour from a second bottle to the chloroform vapour from a Junker's apparatus; and to the address of Dr. Waller on the dosage of anæsthetics, embodying his conclusions from experiments on nerve. He lays stress on the great fact that the lethal dose of chloroform is only twice the anæsthetic dose. The book is sent out in an unattractive dress, and has neither table of contents nor index.

**Annual and Analytical Cyclopædia of Practical Medicine.** Vol. III.—*Dislocations to Infantile Myxœdema.* Philadelphia: The F. A. Davis Company. 1899.—Dr. Sajous, the editor of this stupendous work, states that the kind reception afforded the first two volumes has been the source of much gratification. The plan of the work has met the wants of the general practitioner, while preserving for authors and teachers the leading advantages of the older annual. On reading through several of the articles, *e.g.*, epilepsy, exophthalmic goitre, infantile myxœdema, hypnotism, and hysteria, we have found them to be very accurate and complete monographs on the respective subjects.

**E. Merck's Annual Report [of New Medicinal Preparations] on the year 1898.** Pp. 185. Darmstadt: Eduard Roether. 1899. —The composition and action of many modern drugs are here described, together with an original communication on the action of some morphia derivatives. The well-known name of Merck requires no commendation; but many of these preparations, though interesting from the chemical standpoint, are too much in advance of the Pharmacopœia to be of great utility to other than the experimental therapeutist. Our patients commonly will not be satisfied to spend time and money on drugs whose reputation has not yet borne the test of time.

**Therapeutic Notes.** London: Parke, Davis & Co.—Dated July, this is the first number of a serial which is a simple advertisement, bright, crisp, and up-to-date, of some of the preparations of this enterprising firm.

**The British Physician.** London: The Sanitary Publishing Co., Ltd.—This new monthly journal aims at a more or less distinctive character, and "while admitting contributions on any and every branch of medical science and practice, will be more closely concerned with therapeutics and pharmacology." It will avoid matters of purely surgical interest. We trust that the editor will be able to carry out his views and will have the support of a large staff of scientific contributors, both metropolitan and provincial.

**The Caledonian Medical Journal.** Glasgow: Alex. Macdougall. —This new exchange contains the proceedings of the Caledonian Medical Society—which is peripatetic, holding its annual meetings in various towns. The editors are Dr. Macnaughton of Stonehaven, and Dr. Andrew Little of Burnley, who inform us that the reputation of the *Journal* is steadily increasing, and that no pains will be spared to make it bright, interesting, and readable. If the members of this Society are not immortalised by their professional doings, they will be borne in mind by an amused posterity, for a picture of them at dinner is given as a frontispiece to this number.

**The Edinburgh Medical Journal.** New Series. Vol. V. Edinburgh: Young J. Pentland. 1899.—We have in this publication a nicely printed volume of 648 pages, containing a large number of original articles, many reviews of recent books, and a monthly account of the recent advances in the various departments of medical science. Those who do not see the monthly numbers of the *Edinburgh Medical Journal* would do well to peruse the six-monthly volume, which contains the views of the best authorities on many modern topics.

**A Year's Work in Medical Defence, 1898.** London: 4 Trafalgar Square.—From the annual report of the Medical Defence Union we learn that the condition of the fund is satisfactory, and that the Union continues to gain members. Of its utility, if proof were needed, the present report is evidence. We cannot help regretting that the Council have decided not to proceed further in the prosecution of quacks, although we believe such a duty is one that naturally devolves upon the General Medical Council, but that body seems to prefer to obtain convictions for qualified men. We may call attention to the change of address, so that medical men wishing to join should now write to the address given above.

**Archives of the Roentgen Ray.** Vol. II. London: The Rebman Publishing Company, Limited. 1898.—Vol. ii. of this interesting chronicle of skiagraphical progress, though it has no very remarkable development of its subject either in theory or practice to record, contains nevertheless particulars of a good deal of important work which has been done with the object of enlarging the possibilities and ensuring the success of X-ray diagnosis. Two questions of special practical moment are conspicuously to the fore. The first is the exact localisation of foreign bodies when surrounded by a great thickness of tissue, and this receives a good deal of attention, several methods of procedure, both with the photographic plate and with the fluorescent screen, being described. The other question is the structure and efficiency of that most essential of all the items of skiagraphical equipment—the X-ray tube. This is also dealt with at considerable length, and considering how fickle and untrustworthy, for the most part, the average tube proves when put to the test of serious use, such investigations into the conditions of maximum efficiency as are here recorded are of the highest value. In addition to the letterpress, the volume contains numerous well-executed reproductions of skiagrams. Many of these are interesting, though none present features of special novelty, except perhaps Dr. W. J. Morton's skiagram of the whole of an adult, taken by a single exposure of thirty minutes.

**Burdett's Official Nursing Directory, 1899.** London: The Scientific Press, Limited.—The second year of the *Nursing Directory* differs little from the previous one, which we noticed a year ago. Testing it by reference to the various institutions we know locally and elsewhere, we find it correct, and the information as complete as required. Perhaps we shall some day have the legal status of a nurse defined, and then the *Directory* will really be of "official" value. We still think it desirable that there should be local lists.

**Year-Book of the Scientific and Learned Societies of Great Britain and Ireland.** Sixteenth Annual Issue. London: Charles Griffin and Company, Limited. 1899.—It seems strange that the compilers of this book will not increase its value by providing a more comprehensive index, which should include references to the papers read before the societies. Some of the slight errors in its pages might be removed if a proof was sent to the numerous secretaries of the societies recorded.

**Knowledge.** June, 1899. London: T. Thompson.—We are glad to see this old-established journal showing such sustained vigour. The number of interesting original scientific articles is remarkable, and the illustrations are good. As a result of a careful study of the Hereford earthquake of 1896, Dr. Charles Davison puts forward an ingenious theory of the origin of the double focus in earthquakes. A map shows the large area affected by this disturbance, which acted over probably 100,000 square miles. There are charming papers on certain crustacea which grow to fit the habitations they adopt, and on the organisms to which flint is originally due; while the column of ornithological notes will attract many lovers of rural sights and sounds. A paper by Professor A. Thomson gives us in graphic form the proportions of the limbs and trunk of the human subject in different races. The long arms and long legs of the negro, the shortness of the upper in contrast with the lower limb of the white man, and the long legs and short body of the Australian are strikingly displayed. The ultimate reduction of the great existing mass of anthropological data to some useful form seems to depend on the adoption of some such method as this.

**Physicians' Book for Private Formulæ: with Posological Table.** Bristol: John Wright & Co. [1898.]—Those who have short memories may find this little book useful for recording formulæ, the details of which they have difficulty in carrying in their heads. The blank pages for this purpose, together with the doses of the last *British Pharmacopœia* preparations and much other useful information, make up a tiny but elegant volume which can really be a waistcoat-pocket companion whose society may often be of value to the doctor.

**Programme of the Inauguration. Wellcome Club and Institute, June, 1899.**—The enterprise of the firm of Messrs. Burroughs, Wellcome, & Co. is not limited to providing facilities for the prescribing doctor, but has an ever watchful care for the well-being of those whom it employs. The last practical shape which this has taken is a Club and Institute at Dartford, which, to judge by the illustrations given in the booklet, must be a truly delightful place.

